# Random Effects Multinomial Processing Tree Models: A Maximum Likelihood Approach

**DOI:** 10.1007/s11336-023-09921-w

**Published:** 2023-05-29

**Authors:** Steffen Nestler, Edgar Erdfelder

**Affiliations:** 1 Institut für Psychologie, Universität Münster, Fliednerstr. 21, 48149 Münster, Germany; 2 Universität Mannheim, Fakultät für Sozialwissenschaften A5, 68159 Mannheim, Germany

**Keywords:** multinomial processing tree models, random effects models, hierarchical models, maximum likelihood estimation

## Abstract

The present article proposes and evaluates marginal maximum likelihood (ML) estimation methods for hierarchical multinomial processing tree (MPT) models with random and fixed effects. We assume that an identifiable MPT model with *S* parameters holds for each participant. Of these *S* parameters, *R* parameters are assumed to vary randomly between participants, and the remaining $S-R$ parameters are assumed to be fixed. We also propose an extended version of the model that includes effects of covariates on MPT model parameters. Because the likelihood functions of both versions of the model are too complex to be tractable, we propose three numerical methods to approximate the integrals that occur in the likelihood function, namely, the Laplace approximation (LA), adaptive Gauss–Hermite quadrature (AGHQ), and Quasi Monte Carlo (QMC) integration. We compare these three methods in a simulation study and show that AGHQ performs well in terms of both bias and coverage rate. QMC also performs well but the number of responses per participant must be sufficiently large. In contrast, LA fails quite often due to undefined standard errors. We also suggest ML-based methods to test the goodness of fit and to compare models taking model complexity into account. The article closes with an illustrative empirical application and an outlook on possible extensions and future applications of the proposed ML approach.

Multinomial processing tree (MPT) models are stochastic models for categorical data frequently used in different branches of behavioral science, primarily in cognitive psychology and social cognition research (for overviews and a recent tutorial see Batchelder & Riefer, 1999; Erdfelder et al., 2009, 2020; Hütter & Klauer, 2016; Schmidt et al., 2023). They have been applied to a wide range of phenomena, including, for example, recognition memory (e.g., Riefer & Batchelder, 1995; Xu & Bellezza, 2001), source monitoring (e.g., Meiser & Broder, 2002), recall memory (Batchelder & Riefer, 1986), and judgmental illusions, such as the hindsight bias (Erdfelder and Buchner, 1998; Nestler and Egloff, 2009; Nestler et al., 2012). Specifically, MPT models are cognitive process models that refer to a particular experimental task or paradigm in which participants’ judgments are categorized into a well-defined set of responses. It is assumed that the observed frequencies of responses who fall into these categories follow a multinomial distribution and that the probabilities underlying these frequencies are determined by latent cognitive processes that drive observed response behavior.

The primary goal of fitting MPT models to observed response frequencies is to estimate the cognitive process parameters, that is, the latent probabilities that certain cognitive processes were successful or not (e.g., memory processes, such as encoding, storage, or retrieval). Furthermore, by estimating models that impose psychologically motivated restrictions on these parameters (e.g., equality constraints or parameter fixations), model comparisons can be used to statistically test psychological assumptions and cognitive hypotheses that are linked to the model.

In most past applications, MPT models have been estimated by aggregating observed category frequencies across participants. This approach presumes that individual differences in cognitive process parameters can be neglected. However, when there are substantial individual differences, parameter estimates may be biased, and the results of inferential statistical procedures might not be optimal (e.g., the standard errors of the parameter estimates may be misleading, Batchelder & Riefer, 1999; Erdfelder et al., 2009; Klauer, 2006; 2010; Smith & Batchelder, 2010). In addition to these statistical problems, the assumption that there are no between-person differences in relevant cognitive processes seems rather implausible (Lee & Webb, 2005; Smith & Batchelder, 2010). Such an assumption also precludes the exploration of interesting research questions about the origin of individual differences in process parameters and about their relationships with other model parameters as well as covariates (e.g., Coolin et al., 2015, 2016). Hence, it is desirable to quantify potential interindividual differences and to investigate which person variables can explain them.

A number of extensions have therefore been proposed to model and predict the heterogeneity of parameters between individuals. First, Klauer (2006) suggested a latent class MPT framework in which a single person is assumed to be a member of one latent class, and model parameters may differ between latent classes. Second, Smith and Batchelder (2010) proposed a hierarchical MPT model in which participants’ model parameters are assumed to be sampled from *independent* beta distributions (i.e., a beta distribution for each MPT model parameter; hence the name “beta-MPT model”). More recently, Klauer (2010) introduced another hierarchical extension of the standard MPT model that allows researchers to capture not only the variability in the (probit-transformed) parameters across individuals but also the covariances or correlations between these parameters. This latent-trait MPT model is based on the assumption that the person parameters stem from a multivariate normal distribution, and their expectations and covariance matrix are estimated from the observed frequency data.

Klauer (2010) suggested a Bayesian approach for parameter estimation and inferences in which the posterior distribution is approximated using Monte Carlo-Markov chain methods (see Heck et al., 2018a, for an implementation in R). In the present article, we show how the parameters of the latent-trait MPT model can be obtained through marginal maximum likelihood (ML) estimation. Our implementation of the ML method introduces a frequentist approach for hierarchical MPT models that is perhaps more familiar to researchers because standard errors, confidence intervals, and goodness-of-fit tests can be computed on the basis of well-known asymptotic properties of ML estimates. Moreover, in addition to the asymptotic optimality of ML estimates (Reed and Cressie, 1988), the subtleties of specifying appropriate prior distributions for Bayesian estimation can be avoided when using the ML method. In particular, one does not have to worry about whether and how the obtained estimates and model comparisons are affected by the prior (for a recent discussion, see Sarafoglou et al., 2022) or whether de-facto equivalent models may become nonequivalent as a consequence of the choice of the prior (Kellen & Klauer, 2020). Importantly, the most efficient numerical ML algorithm is also typically faster than a Bayesian estimator, and the convergence of the ML algorithm is simpler to determine.

## The Pair-Clustering Model

**Fig. 1:**
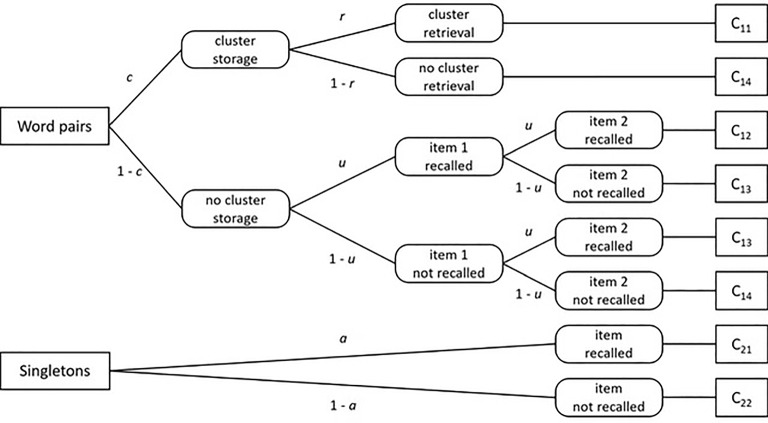
The pair-clustering MPT model (adapted from Riefer & Batchelder, 1988, p. 330, Figure 2). Rectangles indicate stimulus classes (left) and observable responses (right). Rectangles with rounded corners represent latent cognitive states. Parameters attached to the branches indicate transition probabilities from left to right, specifically, storing a word pair as a cluster (*c*), retrieving a stored cluster in free recall (*r*), storing and retrieving a word from a non-clustered pair in free recall (*u*), and storing and retrieving a singleton in free recall (*a*).

Before we introduce MPT models and the ML estimation methods in more detail, we briefly describe the pair-clustering model (e.g., Batchelder & Riefer, 1980, 1986) that we use throughout the article to illustrate the proposed methods. The pair-clustering model can be used to analyze data from a free-recall experiment in which participants are presented with a list of word pairs plus a number of singletons. All words are preselected to be equally difficult in terms of memorizability. Each word pair consists of semantically related words (e.g., rose–tulip), whereas singletons are unrelated to other words in the list. These pairs and the singletons are presented one word at a time, and participants are later asked to recall the items from the list in any order. On the basis of participants’ free recall performance, the studied word pairs and singletons are then assigned to one of six response categories. It is assumed that the probability of each response category can be modeled by two processing trees that include a total of four parameters (see Fig. 1 for a graphical illustration of the model).

Specifically, the model defines four response categories for the word pairs: Category $C_{11}$ includes all cases where both words in a pair are recalled adjacently, $C_{12}$ represents cases in which both words are recalled nonadjacently, $C_{13}$ corresponds to cases where exactly one word is recalled, and finally $C_{14}$ represents cases in which neither word from the pair is recalled. In addition, the singletons are scored in two categories: singletons recalled successfully ($C_{21}$) versus not successfully ($C_{22}$).

The pair-clustering model proposes four cognitive process parameters that jointly determine the response probabilities: (1) the probability *c* that a word pair is stored as a cluster in memory, (2) the probability *r* that a stored cluster is successfully retrieved in free recall, (3) the probability *u* that one word from a pair that is not stored as part of a cluster is successfully retrieved, and finally, (4) the probability *a* that a singleton is successfully stored and retrieved. These parameters can be used to derive response probabilities for each of the six categories by calculating the product of all parameters along a branch and then summing up all branch probabilities that refer to the same category. For example, because there is only one branch that terminates in category $C_{11}$ (see Fig. 1), the probability of this category (i.e., both words recalled adjacently) is just the product $c \cdot r$ (i.e., successful storage as a cluster followed by successful retrieval of this cluster). Applying the same logic to all branches in Fig. 1 results in the following six model equations:

1 $$\begin{aligned} P(C_{11})&= c \cdot r \nonumber \\ P(C_{12})&= (1 - c) \cdot u^2 \nonumber \\ P(C_{13})&= (1 - c) \cdot u \cdot (1 - u) + (1 - c) \cdot (1 - u) \cdot u \nonumber \\ P(C_{14})&= c \cdot (1 - r) + (1 - c) \cdot (1 - u)^2 \nonumber \\ P(C_{21})&= a \nonumber \\ P(C_{22})&= 1 - a \end{aligned}$$

The goal of MPT modeling, applied to the pair-clustering model, is to estimate the four cognitive process parameters and to test reasonable hypotheses about these parameters. One example of such a hypothesis would be that unclustered words from a pair behave like singletons in memory, that is, they are stored and retrieved with the same probability (i.e., $u = a$).

In the next section, we begin with a formal introduction of a typical MPT model without random effects. This is followed by an outline of the latent-trait MPT model with some extensions. In the third section, we present three methods of marginal ML estimation for the latent-trait model. Next, we describe the results of a small simulation study in which we compare the different methods with respect to accuracy and speed. In Sect. 3.2, we present an application of our method to an illustrative example using real data. In the final section, we discuss possible extensions and open questions for future research.

## MPT Models Without Person-Level Random Effects

The pair-clustering model we just described is a specific instance of an MPT model. On a more general level, MPT models are statistical models of response frequencies of mutually exclusive, independent categories. To define the model, we assume that there are $k = 1, \ldots , K$ category systems and that each category system consists of $j = 1$, $\ldots$, $J_k$ categories $C_{kj}$. In the pair-clustering model, there are $K = 2$ category systems, one for the word pairs and one for the singletons. The first system consists of four categories: $C_{11}$, $C_{12}$, $C_{13}$, and $C_{14}$; and the singleton system contains two categories: $C_{21}$ and $C_{22}$. We further assume that data from $t = 1$, $\ldots$, *T* individuals is available. For each single individual *t*, the data for category system *k* are given as a vector of frequencies, $\varvec{n}_{kt} = (n_{k1t}, \ldots , n_{kJ_{k}t})$. For instance, $\varvec{n}_{1t}$ contains the four frequencies of person *t* for the four word pair categories (i.e., *k* = 1). These frequencies are assumed to stem from a multinomial distribution

2 $$\begin{aligned} f(\varvec{n}_{kt}|\varvec{\theta }) = \begin{pmatrix} N_{kt} \\ n_{k1t} ... n_{kJ_{k}t} \end{pmatrix} p_{k1t}^{n_{k1t}} \cdot p_{k2t}^{n_{k2t}} \cdots p_{kJ_{k}t}^{n_{kJ_{k}t}} = \begin{pmatrix} N_{kt} \\ n_{k1t} ... n_{kJ_{k}t} \end{pmatrix} \prod _{j = 1}^{J_{k}} p_{kjt}^{n_{kjt}} \end{aligned}$$

where $N_{kt}$ = $n_{k1t}$ +... + $n_{kJ_{k}t}$ and $p_{k1t} + \cdots + p_{kJ_{k}t} = 1$. This definition requires the vector of all frequencies across all category systems *K* of person *t*, that is, $\varvec{n}_{t} = (\varvec{n}_{1t},..., \varvec{n}_{Kt})$, to follow a product multinomial distribution:

3 $$\begin{aligned} f(\varvec{n}_{t}|\varvec{\theta }) = \prod _{k = 1}^{K} \begin{pmatrix} N_{kt} \\ n_{k1t} ... n_{kJ_{k}t} \end{pmatrix} \prod _{j = 1}^{J_{k}} p_{kjt}^{n_{kjt}} . \end{aligned}$$

The idea behind MPT models is that the probability of the occurrence of an event category can be modeled as a function of the cognitive process parameters. In the standard model (i.e., without person-level random effects), these process parameters $$\varvec{\theta }$$ are unknown constants that do not vary between individuals. Thus, $$ p_{kjt}$$ is written as

4 $$\begin{aligned} p_{kjt} = P(C_{kj} | \varvec{\theta } ) \end{aligned}$$

where $$\varvec{\theta }$$ contains the *S* cognitive process parameters and is thus an element of $$[0, 1]^S$$, $$s = 1,..., S$$. In the example, $$\varvec{\theta } = (c, r, u, a)$$, and the probability of $$C_{14}$$ (see above) is $$p_{14t} = P( C_{14} | \varvec{\theta } ) = c(1-r) + (1-c)(1-u)^2$$. To write $$P( C_{kj} | \varvec{\theta } )$$ more generally, we define $$ I_{kj}$$ to be the number of branches of the tree that terminate in $$C_{kj}$$, with $$i = 1,..., I_{kj}$$ indexing a specific branch $$B_{kji}$$ that terminates in category *j* of category system *k*. As described by Hu and Batchelder (1994), we define:

5 $$\begin{aligned} P( B_{kji} | \varvec{\theta }) = \prod _{s = 1}^{S} \theta _{s}^{a_{skji}}( 1 -\theta _{s})^{b_{skji}} \end{aligned}$$

where $a_{skji}$ and $b_{skji}$ indicate how often a process parameter $\theta _{s}$ and its complement $1-\theta _{s}$, respectively, appear on the branch $B_{kji}$. For instance, there are two paths that terminate in $C_{14}$. To illustrate, we just consider the first of these branches, $B_{141}$:

6 $$\begin{aligned} P( B_{141} | \varvec{\theta })&= \prod _{s = 1}^{4} \theta _{s}^{a_{s141}}( 1 -\theta _{s})^{b_{s141}} \nonumber \\&= \theta _{1}^{a_{1141}}( 1 -\theta _{1})^{b_{1141}} \cdot \theta _{2}^{a_{2141}}( 1 -\theta _{2})^{b_{2141}} \cdot \theta _{3}^{a_{3141}}( 1 -\theta _{3})^{b_{3141}} \cdot \theta _{4}^{a_{4141}}( 1 -\theta _{4})^{b_{1141}}\nonumber \\&= c^{1}(1-c)^{0} \cdot r^{0}(1-r)^{1} \cdot u^{0}(1-u)^{0} \cdot a^{0}(1-a)^{0} = c(1-r) \end{aligned}$$

According to these definitions, the probability of $C_{kj}$ is

7 $$\begin{aligned} P(C_{kj} | \varvec{\theta } ) = \sum _{i = 1}^{I_k} P( B_{ kji} | \varvec{\theta }) = \sum _{i = 1}^{I_k} \prod _{s = 1}^{S} \theta _{s}^{a_{skji}}( 1 -\theta _{s})^{b_{skji}} \end{aligned}$$

and the multinomial probability of the data for a single individual *t* is

8 $$\begin{aligned} f(\varvec{n}_{t}|\varvec{\theta }) = \prod _{k = 1}^{K} \begin{pmatrix} N_{kt} \\ n_{k1t} ... n_{kJ_{k}t} \end{pmatrix} \prod _{j = 1}^{J_{k}} \left[ \sum _{i = 1}^{I_{kj}} \prod _{s = 1}^{S} \theta _{s}^{a_{skji}}( 1 -\theta _{s})^{b_{skji}} \right] ^{n_{kjt}}. \end{aligned}$$

Equation 8 can be used to obtain ML estimates of the process parameters $\varvec{\theta }$. For instance, Hu and Batchelder (1994) showed how the parameters can be estimated with an EM algorithm. Alternatively, methods based on the analytical gradient or finite difference methods can be used for ML parameter estimation. Both approaches are implemented in the R package MPTinR (Singmann and Kellen, 2013; Singmann et al., 2020) or the package mpt (Wickelmaier and Zeileis, 2011, 2020). Also, a Bayesian approach can be used to estimate the parameters (e.g., Lee & Wagenmakers, 2014).

## MPT Models with Person-Level Random Effects

In the previous section, the process parameters contained in $\varvec{\theta }$ were assumed not to differ between individuals. In the latent-trait MPT model, this assumption is relaxed because $R {\le } S$ elements of $\varvec{\theta }$ are written as a function of person-specific random effects that stem from a multivariate normal distribution

9 $$\begin{aligned} \varvec{b}_t \sim MNV( \varvec{\mu }, \varvec{\Sigma }) \end{aligned}$$

where $\varvec{\mu }$ is an $R \times 1$ vector of expectations and $\varvec{\Sigma }$ is an $R \times R$ covariance matrix. Because each single $\theta _{st}$ is a probability parameter, we need to make sure that the resulting coefficient remains in the unit interval [0, 1]. On the basis of the literature on the Generalized Linear Model (GLM), we use the logit-link function (see Coolin et al., 2015)

10 $$\begin{aligned} \theta _{st} = \frac{1}{1 + {exp}\left[ -(\beta _s + b_{st})\right] } \end{aligned}$$

or the probit-link function (see Klauer, 2010)

11 $$\begin{aligned} \theta _{st} = \Phi (\beta _s + b_{st}) \end{aligned}$$

to map a real-valued person parameter $b_{st}$ on a probability parameter $\theta _{st}$, with $\beta _s$ serving as a parameter-specific intercept constant (in Eq. 11, $\Phi$ denotes the cumulative normal distribution). The two approaches typically result in very similar parameter estimates (at least in the GLM framework). To allow for comparisons in the framework of random effects MPT models, we have implemented both link functions in the R package that implements the statistical methods proposed in this article.

We can now define the *conditional* distribution of the category frequencies of person *t* given the random effects $\varvec{b}_t$:

12 $$\begin{aligned} f(\varvec{n}_{t}|\varvec{\beta }, \varvec{b}_t) = \prod _{k = 1}^{K} \begin{pmatrix} N_{kt} \\ n_{k1t} ... n_{kJ_{k}t} \end{pmatrix} \prod _{j = 1}^{J_{k}} \left[ \sum _{i = 1}^{I_{kj}} \prod _{s = 1}^{S} \theta _{st}^{a_{skji}}( 1 -\theta _{st})^{b_{skji}} \right] ^{n_{kjt}} \end{aligned}$$

where $\theta _{st}$ is defined as in Eqs. 10 or 11. Along with the multivariate normal density of the random effects $\varvec{b}_t$, Eq. 12 can then be used to obtain ML estimates of the model parameters. We refer to this model as the random effects MPT model.

Before we proceed, we would like to point out that the mean structure in our model is not identified because the expectation of the random effects is $\varvec{\mu }$ and the conditional distribution contains the vector of intercept terms $\varvec{\beta }$. To identify the mean structure, we define all elements of $\varvec{\beta }$ to be zero when *all* process parameters are assumed to differ between individuals (i.e., $R=S$, where *R* denotes the number of random effects parameters). In this case, our random effects model corresponds to the model described by Klauer (2010). However, when only some (but not all) of the process parameters are assumed to be random, we estimate the respective entries in $\varvec{\beta }$ for the $(S-R)$ fixed parameters while setting the remaining *R* entries (corresponding to random parameters) to zero and reducing the dimensions of $\varvec{\mu }$ and $\varvec{\Sigma }$ accordingly. Thus, by writing the model as proposed here, we achieve a high degree of flexibility to estimate MPT models with both random and fixed effects. This can be very useful, for example, when MPT models include guessing parameters that need to be estimated and are assumed to be constant across individuals.

Furthermore, we can also extend the model to include person-level covariates contained in the person-specific column vectors $\varvec{X}_{t}$. This can be achieved in two ways: First, when the person-level covariates are used to predict *R* process parameters that are assumed to be random, $\varvec{\mu }$ is person-specific, and we write it as function of the predictor values:

13 $$\begin{aligned} \varvec{\mu }_t = \varvec{\mu } + \varvec{\Gamma }\varvec{X}_{t}, \end{aligned}$$

where $\varvec{\Gamma }$ is an $R \times p$ matrix of weights, and *p* is the number of person-level predictors used to predict the random process parameters. Note that when there is an assumption that a process parameter is *not* affected by a predictor in $\varvec{X}_{t}$, the respective entry in $\varvec{\Gamma }$ is simply set to zero. Furthermore, $\varvec{\mu }$ represents the values of the person parameters when the covariates are zero. Second, when a fixed process parameter $\theta _s$ is assumed to vary as a function of person-level covariates so that the intercept $\beta _{st}$ may differ between individuals *t*, we follow the procedure outlined by Coolin et al. (2015) and write

14 $$\begin{aligned} \beta _{st} = \beta _{s} + \varvec{\gamma _{s} } \varvec{X}_{t} \end{aligned}$$

where $\beta _{s}$ is the value of the parameter-specific intercept when all person-level covariates are zero and $\varvec{\gamma _{s}}$ is a row vector containing the weights of the *p* covariates used to predict $\theta _s$.

### Marginal Maximum Likelihood Estimation

The goal is to estimate the free parameters contained in $\varvec{\beta }$, $\varvec{\gamma }$, $\varvec{\mu }$, $\varvec{\Gamma }$, and $\varvec{\Sigma }$, where $\varvec{\beta }$ and $\varvec{\gamma }$ denote the $(S-R)$-dimensional vector of intercepts and the $(S-R) \times p$ matrix of predictor weights, respectively, in the fixed-effects part of our model (corresponding to the model of Coolin et al., 2015) while $\varvec{\mu }$, $\varvec{\Gamma }$, and $\varvec{\Sigma }$ denote to the *R*-dimensional vector of parameter means, the $R \times p$ matrix of predictor weights, and the $R \times R$ parameter covariance matrix, respectively, in the random-effects part of our model (corresponding to the model of Klauer, 2010). We employ a marginal ML approach that is based on the marginal density of the response frequencies. For a single individual *t*, this density is

15 $$\begin{aligned} f(\varvec{n}_{t}) = \int _{\varvec{b}_t} f(\varvec{n}_{t}|\varvec{\beta },\varvec{\gamma }, \varvec{b}_t) f(\varvec{b}_t|\varvec{\mu },\varvec{\Gamma },\varvec{\Sigma }) {d}\varvec{b}_t . \end{aligned}$$

and for the entire sample, it is

16 $$\begin{aligned} f(\varvec{n}) = \prod _{t = 1}^{T} f(\varvec{n}_t) \end{aligned}$$

Eq. 16 can be used to define the likelihood

17 $$\begin{aligned} L(\varvec{\beta },\varvec{\gamma },\varvec{\mu },\varvec{\Gamma },\varvec{\Sigma }) = \prod _{t = 1}^{T} \int _{\varvec{b}_t} f(\varvec{n}_{t}|\varvec{\beta },\varvec{\gamma }, \varvec{b}_t) f(\varvec{b}_t|\varvec{\mu },\varvec{\Gamma },\varvec{\Sigma }) {d}\varvec{b}_t = \prod _{t = 1}^{T} \int _{\varvec{b}_t} L_t {d}\varvec{b}_t \end{aligned}$$

and the log-likelihood of the data

18 $$\begin{aligned} ll(\varvec{\beta },\varvec{\gamma }, \varvec{\mu },\varvec{\Gamma },\varvec{\Sigma }) = \sum _{t = 1}^{T} \text {log}\left( \int _{\varvec{b}_t} L_t {d}\varvec{b}_t\right) . \end{aligned}$$

One problem with the marginal ML approach is that there is no analytical solution for the values of $\varvec{\beta }$, $\varvec{\gamma }$, $\varvec{\mu }$, $\varvec{\Gamma }$, and $\varvec{\Sigma }$ that maximize Eq. 18. Rather, numerical approximations have to be used to estimate the model parameters. For these approximations, we use the analytical gradient of the log-likelihood function given by

19 $$\begin{aligned} \frac{\partial ll}{\partial \tau _k} = \sum _{t = 1}^{T} \frac{1}{ f (\varvec{n}_{t}) } \int _{\varvec{b}_t} L_t \frac{\partial \text {log}(L_t)}{\partial \tau _k} {d}\varvec{b}_t \end{aligned}$$

where $\tau$ denotes an element of $\varvec{\beta }$, $\varvec{\gamma }$, $\varvec{\mu }$, $\varvec{\Gamma }$, or $\varvec{\Sigma }$. To implement the gradient, one needs the first derivative of the log-likelihood function for a single person *t* with regard to a parameter. Note that $\text {log}(L_t)$ is

20 $$\begin{aligned} \text {log}f\left( \varvec{n}_{t}|\varvec{\beta },\varvec{\gamma }, \varvec{b}_t\right) + \text {log}f \left( \varvec{b}_t|\varvec{\mu }, \varvec{\Gamma }, \varvec{\Sigma }\right) \end{aligned}$$

Thus, for a single element $\sigma _{l}$ contained in $\varvec{\Sigma }$, the derivative is

21 $$\begin{aligned} \frac{\partial \text {log}L_t}{\partial \sigma _l} = -\frac{1}{2} tr\left( \varvec{\Sigma }^{-1} \frac{\partial \varvec{\Sigma }}{ \partial \sigma _l }\right) + \frac{1}{2} \left( \varvec{b}_{t} - \varvec{\mu }_{t}\right) ^{'} \varvec{\Sigma }^{-1} \frac{\partial \varvec{\Sigma }}{ \partial \sigma _l } \varvec{\Sigma }^{-1} (\varvec{b}_{t} - \varvec{\mu }_{t}) \end{aligned}$$

with

22 $$\begin{aligned} \frac{\partial \varvec{\Sigma }}{ \partial \sigma _l } = \varvec{1}_{l} + \varvec{1}_{l}^{'} - \varvec{1}_{l} \cdot \varvec{1}_{l}^{'} \end{aligned}$$

and $\varvec{1}_{l}$ is an *R* x *R* matrix that contains a 1 in the position of $\sigma _l$ and zeroes in all other positions. For elements in $\varvec{\mu }$ or $\varvec{\Gamma }$, the derivative is

23 $$\begin{aligned} \frac{\partial \text {log}L_t}{\partial \mu _{tl}} = (\varvec{b}_t - \varvec{\mu }_t)^{'} \varvec{\Sigma }^{-1} \frac{\partial \varvec{\mu }_{t}}{ \partial \mu _{tl} } \end{aligned}$$

where

24 $$\begin{aligned} \frac{\varvec{\mu }_{t}}{ \partial \mu _l} = \varvec{1}_l \text { and } \frac{\varvec{\mu }_{t}}{ \partial \gamma _l} = \varvec{1}_l \varvec{X}_t \end{aligned}$$

and $\varvec{1}_{l}$ is an *R* x 1 vector or an *R* x *p* matrix that contains a 1 in the position of the parameter that is being estimated and zeroes in all other positions. Finally, the derivative of $\text {log}(L_t)$ with regard to the elements in $\varvec{\beta }$ is

25 $$\begin{aligned} \frac{\partial \text {log}L_t}{\partial \beta _{l}} \sum _{t=1}^{T} \sum _{k=1}^{K} \sum _{j=1}^{J_{k}} \frac{ n_{kjt} }{ P(C_{kj} | \varvec{\theta }_t ) } \sum _{i = 1}^{I_{kj}} \left[ a_{skji} \cdot \left( \frac{\partial \theta _{st}}{\partial \beta _{l}}\right) ^{-1} - b_{skji} \cdot {\left( 1 - \frac{\partial \theta _{st}}{\partial \beta _{l}}\right) }^{-1} \right] \cdot P(B_{kji}|\varvec{\theta }_t) \end{aligned}$$

where $\frac{\partial \theta _{st}}{\partial \beta _{l}}$ is the derivative of the chosen link function with respect to the intercept parameter $\beta _{l}$. The same formula can be used for the parameters in $\varvec{\gamma }$, but the derivative of the link function has to be computed for the element $\gamma _{sl}$ rather than $\beta _{l}$ (i.e., $\frac{\partial \theta _{st}}{\partial \gamma _{sl}}$).

A second problem with using the marginal ML approach is that the likelihood or log-likelihood function, respectively, and the gradient involve integrals that are not tractable. However, one can approximate the integrals using a number of numerical techniques (see Tuerlinckx et al., 2006; Nestler, 2020, 2021). In the R package that implements marginal ML estimation methods, we implemented three approaches: the Laplace approximation, the Adaptive Gauss–Hermite Quadrature (AGHQ), and Quasi Monte Carlo (QMC) Sampling.

***Laplace Approximation*** The basic idea behind the Laplace approximation is that it can be used to replace the function within the integral with another function that has a closed-form expression. Imagine that the modes of the random effects $\varvec{b}_t$ of

26 $$\begin{aligned} l( \varvec{b}_t) = f(\varvec{n}_{t}|\varvec{\beta }, \varvec{\gamma }, \varvec{b}_t) f(\varvec{b}_t|\varvec{\mu },\varvec{\Gamma },\varvec{\Sigma }) \end{aligned}$$

are available for each of the *T* individuals. One can then show (see, e.g., Pinheiro & Bates, 1995) that the likelihood function given in Eq. 17 can be approximated by

27 $$\begin{aligned} L(\varvec{\beta },\varvec{\gamma },\varvec{\mu },\varvec{\Gamma },\varvec{\Sigma }) \approx \prod _{t = 1}^{T} (2\pi )^{S/2}|\hat{\varvec{\Omega }}|^{1/2} f(\varvec{n}_{t}|\varvec{\beta }, \varvec{\gamma }, \hat{\varvec{b}}_t) f( \hat{\varvec{b}}_t|\varvec{\Sigma },\varvec{\mu },\varvec{\Gamma }) \end{aligned}$$

where $\hat{\varvec{b}_t}$ denotes the modes, and $\hat{\varvec{\Omega }}$ is the asymptotic covariance matrix of these modes.

A problem with using Eq. 27 is that one has to estimate the modes $\hat{\varvec{b}_t}$ for all *T* individuals given the (unknown) parameters contained in $\varvec{\beta }$, $\varvec{\gamma }$, $\varvec{\mu }$, $\varvec{\Gamma }$, and $\varvec{\Sigma }$. In practical implementations, this problem is circumvented by first estimating the modes given the current parameter estimates. Then, the model parameters are estimated by maximizing Eq. 27 given the current modes $\hat{\varvec{b}}_t$. This two-step procedure is repeated until the algorithm converges. The Laplace approximation is very fast (compared with the other methods), and, in the well-known R package lme4 (Bates et al., 2015), it is the default method for estimating the parameters of a Generalized Linear Mixed Model (GLMM).

***Gauss–Hermite Quadrature*** The basic idea behind quadrature approaches is that they can be used to approximate the numerical value of the integral. When one assumes that the random effects are normally distributed (as we did), one first generates *M* vectors of size *R* x 1 of Gauss–Hermite (GH) nodes $\varvec{x}$ and weights $\varvec{w}$. For each of the node vectors (e.g., $\varvec{x}_m$), one then computes $f(\varvec{n}_{t}|\varvec{\beta }, \varvec{\gamma }, \varvec{b}_t = \varvec{x}_m )$. The integral for person *t* can then be approximated by a weighted sum

28 $$\begin{aligned} L_{t}(\varvec{\beta },\varvec{\gamma },\varvec{\mu },\varvec{\Gamma },\varvec{\Sigma }) \approx \sum _{n_{1} = 1}^{M} \cdots \sum _{n_{S} = 1}^{M} f(\varvec{n}_{t}|\varvec{\beta }, \varvec{\gamma }, \varvec{b}_{n_{1} \cdots n_{R}} ) \varvec{w}_{n_{1}} \cdots \varvec{w}_{n_{R}} . \end{aligned}$$

The main problem with this GH quadrature is that the number of points *M* increases exponentially with the size of *R*. For example, when *M* = 10 and *R* = 4, there are $10^4 = 1000$ single node vectors. This is problematic because $f(\varvec{n}_{t}|\varvec{\beta }, \varvec{\gamma }, \varvec{b}_t = \varvec{x}_m )$ needs to be computed for each vector $\varvec{x}_m$ *and* each person *t*. Thus, when *M* is large, the computational burden associated with approximating the integral is too large to be feasible. For instance, when *M* = 10 and *R* = 10, one would need to compute $10^{10}$ vector values for each person *t*. For this reason, we use Adaptive GH quadrature in our implementation (AGHQ, Rabe-Hesketh et al., 2002; Tuerlinckx et al., 2006). In AGHQ, the GH quadrature points $\varvec{x}$ for each individual are first centered and scaled with the individual’s modes $\hat{\varvec{b}}_t$. These scaled nodes and weights are then used to compute a weighted sum that is similar to the one provided in Eq. 28. By scaling the node vectors with the modes, fewer nodes are required to achieve a precise approximation of the integrals.

***Quasi Monte Carlo Integration*** Even when using AGHQ, the computational burden is too high for high-dimensional random effect distributions. An alternative one could use is Monte Carlo (MC) integration. MC integration rests on the observation that the integral in Eq. 15 can be seen as an expectation of the function $f(\varvec{n}_{t}|\varvec{\beta },\varvec{\gamma }, \varvec{b}_t)$ with respect to the random effect distribution $f(\varvec{b}_t|\varvec{\mu },\varvec{\Gamma },\varvec{\Sigma })$:

29 $$\begin{aligned} \int _{\varvec{b}_t} f(\varvec{n}_{t}|\varvec{\beta }, \varvec{\gamma }, \varvec{b}_t) f(\varvec{b}_t|\varvec{\mu },\varvec{\Gamma },\varvec{\Sigma }) {d}\varvec{b}_t = \text {E} \left[ f(\varvec{n}_{t}|\varvec{\beta }, \varvec{\gamma }, \varvec{b}_t) \right] . \end{aligned}$$

One can thus draw *M* random samples from $f(\varvec{b}_t|\varvec{\mu },\varvec{\Gamma },\varvec{\Sigma })$ to approximate the integral (Robert and Casella, 2010) with

30 $$\begin{aligned} L_{t}(\varvec{\beta },\varvec{\gamma }, \varvec{\mu },\varvec{\Gamma },\varvec{\Sigma }) \approx \frac{1}{M} \sum _{m = 1}^{M} f(\varvec{n}_{t}|\varvec{\beta },\varvec{\gamma }, \varvec{b}_{tm}) . \end{aligned}$$

where one inserts the *m*-th random draw for $\varvec{b}_{t}$. The advantage of MC integration is that the number of draws *M* does not have to increase when another random effect is added. However, one disadvantage of the technique is that *M* must be sufficiently large to be precise. Furthermore, because samples are randomly drawn from the random effect distribution, a (Monte Carlo) sampling error is introduced into the estimation. For these two reasons, here, we use Quasi Monte Carlo (QMC) integration, which builds on deterministic sequences of points instead of randomly drawn points in MC integration (hence the name “Quasi”). There are different ways to generate such sequences (see González et al., 2006, for an introduction). In accordance with the GLMM literature (e.g., Crowther, 2017; González et al., 2006), we use Halton sequences in our implementation because smaller numbers of draws *M* are required to achieve a precise approximation. To further decrease the computational burden, we additionally scale the Halton numbers with $\hat{\varvec{b}_t}$ and $\hat{\Omega }$.

### Standard Errors, Goodness-of-Fit Tests, and Random Effects

Once the ML estimates have been determined, the estimates and their corresponding standard errors can be used to compute *z* statistics and confidence intervals. From standard ML theory, it follows that the parameters are asymptotically normally distributed with a covariance matrix that is obtained by calculating the inverse of the information matrix. This matrix is the negative of the matrix of second derivatives that is given by

31 $$\begin{aligned} \frac{\partial ll}{\partial \tau _k\tau _j} = \sum _{t = 1}^{T} \frac{1}{ f(\varvec{n}_{t})^2 } \left[ f(\varvec{n}_{t}) \frac{\partial d_t}{\partial \tau _j} - d_{t} d^{T}_{t} \right] \end{aligned}$$

where

32 $$\begin{aligned} d_{t} = \int _{\varvec{b}_t} L_t \frac{\partial \text {log}(L_t)}{\partial \tau _k} {d}\varvec{b}_t \end{aligned}$$

and

33 $$\begin{aligned} \frac{\partial d_t}{\partial \tau _j} = \int _{\varvec{b}_t} \left[ \frac{\partial ^2 \text {log}(L_t)}{\partial \tau _k \tau _j} + \frac{\partial \text {log}(L_t)}{\partial \tau _k} \frac{\partial \text {log}(L_t)}{\partial \tau _j} \right] L_t {d}\varvec{b}_t \end{aligned}$$

Again, due to the integrals involved in Eqs. 32 and 33, the matrix of second derivatives is hard to compute. In our implementation of the ML approach users can therefore choose whether standard errors are based on a numerical approximation of the Hessian with finite-difference methods using the analytical gradient (see Eq. 19) or on the exact hessian computed with Eq. 31.

Furthermore, before researchers interpret MPT parameter estimates, they need to show that their model actually fits the observed data. In addition, researchers very often want to compare the fit of a current model with the fit of a more restricted model that imposes psychologically motivated constraints on the parameters (e.g., equality constraints or parameter fixations). Goodness-of-fit tests and model comparisons can both be conducted by means of a likelihood ratio test

34 $$\begin{aligned} LR = -2\cdot ( ll_r - ll_u ) \end{aligned}$$

where $ll_r$ ($ll_u$) is the log-likelihood value of the restricted (unrestricted) model. Under certain regularity conditions (see Reed & Cressie, 1988), the *LR* test statistic asymptotically follows a central $\chi ^2$ distribution with degrees of freedom equal to the difference between the parameters of the unrestricted and restricted models if the more restricted model actually holds. The likelihood ratio test can be used to compare the fit of two models that differ in the mean structure (e.g., *u* = *a* in the pair-clustering model) or in the covariance structure (e.g., $\sigma _{ua}$ = 0).

The likelihood ratio test is based on the assumption that the models being compared are nested. For non-nested model comparisons, we suggest using the Akaike or the Bayesian Information Criterion (AIC or BIC, respectively):

35 $$\begin{aligned} AIC&= -2 \cdot ll + 2 \cdot df \nonumber \\ BIC&= -2 \cdot ll + \text {log}(n)\cdot df, \end{aligned}$$

where $n = \sum _{t=1}^{T} n_t$ and $df = R + (S - R) + p_{\varvec{\Gamma }} + p_{\varvec{\gamma }} + \text {dim}(\varvec{\Sigma })$. Here, *R* is the number of estimated cognitive process parameters set to be random (i.e., parameters in $\varvec{\mu }$), $S - R$ is the number of fixed cognitive process parameters (i.e., parameters in $\varvec{\beta }$), $p_{\varvec{\Gamma }} + p_{\varvec{\gamma }}$ gives the total number of to-be-estimated weights of the person-level covariates in the random and the fixed effects part of the model (i.e., parameters in $\varvec{\Gamma }$ and $\varvec{\gamma }$), respectively, and $\text {dim}(\varvec{\Sigma })$ represents the number of estimated covariance parameters (see Bates et al., 2015).

Finally, it can also be of interest to obtain estimates of the individual random effects for each participant *t*. As a random effects estimator, $\hat{\varvec{b}}_t$, it is possible to use a participant’s mode, which can be obtained by maximizing Eq. 26, while treating the final model parameter estimates as fixed. Fortunately, these values are already required when estimating the model so that they do not have to be estimated again. Another choice would be the empirical Bayes estimator (Bock and Aitken, 1981):

36 $$\begin{aligned} \hat{b}_{t} = \prod _{t = 1}^{T} \frac{1}{ f(\varvec{n}_{t}) } \int _{\varvec{b}_t} \varvec{b}_t \text {log}(f(\varvec{n}_{t}|\varvec{\beta },\varvec{\gamma }, \varvec{b}_t)) f(\varvec{b}_t|\varvec{\mu },\varvec{\Gamma },\varvec{\Sigma }) {d}\varvec{b}_t . \end{aligned}$$

In this approach, we approximate the integrals using one of the integral approximation methods described above.

## Simulation Study

We performed a simulation study to assess the frequentist properties of the suggested ML estimation approaches and also compare it with the performance of a Bayesian approach. Specifically, we examined the effect of the number of participants and the number of responses per participant on the bias of the parameter estimates and the coverage rate of the corresponding confidence or credibility intervals, respectively. In addition, we examined for the ML estimator how the number of quadrature points in the AGHQ approach and the size of the Halton sequence in the QMC method affect the adequacy of the model parameter estimates.

***Population Model and Simulation Conditions.*** We used the pair-clustering model for this simulation. This MPT model comprises two category systems, including four and two categories, respectively, with response probabilities described by four process parameters *c*, *r*, *u*, and *a*. In our simulation study, the population covariance matrix of these four parameters (describing how they vary across participants) was set to

37 $$\begin{aligned} \varvec{\Sigma } = \begin{pmatrix} 0.50 &{}\quad 0.08 &{}\quad 0.04 &{}\quad 0.00 \\ 0.08 &{}\quad 0.35 &{}\quad 0.03 &{}\quad 0.00 \\ 0.04 &{}\quad 0.03 &{}\quad 0.20 &{}\quad 0.07 \\ 0.00 &{}\quad 0.00 &{}\quad 0.07 &{}\quad 0.20 \\ \end{pmatrix} \end{aligned}$$

where the non-zero covariance terms reflect correlations of 0.30, 0.20, and 0.10. The mean values of the parameters were set to $\mu _{c} = 0.50$, $\mu _{r} = 0.40$, $\mu _{u} = 0.25$, and $\mu _{a} = 0.15$ (see Batchelder & Riefer, 1986).

We manipulated the number of simulated participants (75 vs. 125) and the number of simulated responses per participant (25 vs. 75 vs. 125). In accordance with Batchelder and Riefer (1986), about 80% of the responses were assigned to the first category system (i.e., 20 vs. 60 vs. 100), leaving 20% for the second system (i.e., 5 vs. 15 vs. 25). The R package mvtnorm and the function rmultinorm were used to generate the samples. We drew 500 samples from the population for each of the six simulation conditions.

***Estimators*** All the functions that were required to estimate the parameters with ML were implemented in R (R Core Team, 2020; a working version of the package can be downloaded from https://osf.io/w97m5/). To examine the properties of the ML estimation procedures, the number of quadrature points in the AGHQ method was set to 3, 4, or 5, resulting in $4^3 = 64$, $4^4 = 256$ or $4^5 = 1,024$ node vectors per participant. For the QMC method, the size of the Halton sequence was set to 500, 1000, or 2000. To obtain Bayesian estimates, we employed the TreeBUGS package (Heck et al., 2018a). TreeBUGS uses the JAGS-MCMC sampler (Plummer, 2003) to approximate the posterior distribution of the model’s parameters. For each replication, we fitted the model with the traitMPT function using the default settings. That is, weakly informative priors were specified for all parameters (i.e., normal distributions for the means and a scaled Wishart distributions for the covariance matrix). Furthermore, three chains of 20,000 samples were generated from the posterior distributions, whereby the first 2000 samples were discarded for parameter estimation (i.e., burn-in period). Since TreeBUGS by default provides the means of the posterior distributions as parameter estimates, we decided to used them as the Bayes estimates.

***Dependent Measures*** We used the relative bias (RB) of the parameter estimates and the coverage rate (CR) to investigate the statistical performances of the ML approaches and the Bayes estimator. For the relative bias, we first computed the average parameter estimate in a simulation condition. We then computed the difference between this average and the true parameter and thereafter divided the difference by the true parameter. We consider relative biases below 10% to be acceptable, biases of 10–20% to be substantial, and biases above 20% to be unacceptable (e.g., Forero et al., 2009; Morris et al., 2019). For ML, the confidence interval with the standard error^1^ of an estimate was computed in each replication to determine the observed coverage of the 95% confidence intervals. The coverage was then coded 1 if the true parameter value was included in the interval and 0 if the true parameter was not. We used the same approach to determine the observed coverage of the 95% credibility intervals, but employed the 95% credibility intervals as provided by TreeBUGS as the basis for the coding.

**Table 1:** Relative frequencies of converged replications (CR) and average computation time (in s), depending on the estimator, the type of ML approximation method, the number of individuals *T*, and the number of responses *N* per individual.

Variable	*T*	*N*	Bayes	Laplace	ML-AGHQ			ML-QMC		
					3	4	5	500	1000	2000
CR	75	25	0.56	0.01	0.68	0.84	0.85	0.56	0.61	0.62
		75	0.90	0.38	0.75	0.89	0.84	0.83	0.86	0.89
		150	0.95	0.64	0.95	0.99	0.98	0.96	0.97	0.98
	125	25	0.66	0.01	0.71	0.88	0.89	0.61	0.62	0.64
		75	0.91	0.33	0.90	0.98	0.95	0.88	0.93	0.96
		150	0.94	0.65	1.00	1.00	1.00	1.00	1.00	1.00
Time	75	25	53.53	4.29	12.35	18.78	31.44	25.76	36.18	57.50
		75	53.65	6.73	15.64	22.46	31.46	32.52	42.65	69.05
		150	54.38	7.72	12.60	19.50	29.35	23.75	32.63	54.57
	125	25	85.46	8.52	17.63	27.46	49.13	56.91	83.99	106.4
		75	86.84	10.78	30.60	35.69	41.09	70.24	88.03	111.9
		150	88.18	12.65	26.98	32.92	40.13	46.21	61.35	87.32

AGHQ = Adaptive Gauss–Hermit quadrature with 3, 4, or 5 nodes; QMC = Quasi Monte Carlo integration with 500, 1000, or 2000 points. In case of Bayes, convergence rates were calculated using $\hat{R}$ values. Computation times were determined on an Intel Core i7-6700 with four cores and 16GB RAM.

***Results*** We first examined the percentage of samples in which the estimation algorithm converged for the two estimators, the different approximation methods, the different numbers of simulated participants, and the different numbers of simulated responses per participant. In case of ML, a sample was counted as converged when there were neither inadmissible estimates nor undefined standard errors in the final solution. For Bayes, judging the convergence is a more difficult issue (Hoff, 2009). Here, we decided to use $\hat{R}$ > 1.05 as the criterion, because it can be used well in simulation studies and it is suggested in the literature (e.g., Lynch, 2007). However, we acknowledge that a more or less stringent cutoff may lead to different results and that this should be taken into account in the interpretation of our findings.

As can be seen in Table 1, convergence rates for both estimators increased with the number of participants and the number of responses. In case of ML, the larger the number of points used by a method to approximate the integrals, the better the convergence of the ML estimator. When the number of responses was 75 or 125, convergence rates were acceptable for the AGHQ and QMC methods. In conditions with 25 responses, convergence rates were highest for conditions with the largest size of points. In this case, convergence rates were also similar to the convergence rate of the Bayes estimator. With regard to the Laplace approximation, we found that convergence rates were always below 70% (and even near 0% when number of responses was 25). Further analyses showed that all convergence failures occurred because some (or all) of the standard errors were undefined for the final estimates. These estimates were also very biased. We think that this bias can be explained by noting that the precision of the Laplace approximation depends on whether the shape of the function that is being integrated resembles a multivariate normal distribution. Hence, the method does not perform well for highly non-normal cases (e.g., when the responses are Bernoulli distributed; see Engel, 1998), and we suspect that similar performance problems occur for the MPT model. Finally, Table 1 also contains the average run times of the different methods. We note that comparing the computation times across ML and Bayes is difficult, because they depend on how the approaches are implemented in R (i.e., using C++ in the background or parallelization), how many chains are generated etc. In case of Bayes, all methods were slower for larger numbers of participants. Similarly, ML methods became slower the larger numbers of points used to approximate the integrals and the larger the number of participants.

**Table 2:** Relative bias of parameter estimates in percent, depending on the approximation method, the number of individuals *T*, and the number of responses *N* per individual.

Method	No. Points	Parameter	*T* =75			*T* = 125		
			*N* = 25	*N* = 75	*N* = 125	*N* = 25	*N* = 75	*N* = 125
AGHQ	$4^3$	$\mu$	2.72	1.35	0.36	$-$ 1.07	$-$ 0.34	0.74
		$\sigma ^{2}_{b}$	$-$ 23.6	$-$ 1.62	$-$ 2.94	$-$ 12.4	$-$ 3.86	$-$ 3.53
		$\sigma _{bb}$	12.5	11.8	7.42	15.0	9.91	3.13
	$4^4$	$\mu$	2.11	1.34	0.60	0.27	$-$ 0.59	0.71
		$\sigma ^{2}_{b}$	8.44	0.41	$-$ 1.85	$-$ 1.31	$-$ 1.26	$-$ 2.05
		$\sigma _{bb}$	$-$ 13.8	1.76	3.76	4.53	2.04	$-$ 1.20
	$4^5$	$\mu$	3.99	1.98	0.70	1.71	$-$ 0.79	0.69
		$\sigma ^{2}_{b}$	7.38	2.29	$-$ 1.11	1.28	0.02	$-$ 1.45
		$\sigma _{bb}$	$-$ 8.65	1.59	3.59	$-$ 1.30	$-$ 0.05	$-$ 2.39
QMC	500	$\mu$	$-$ 5.64	$-$ 2.65	$-$ 1.59	$-$ 6.71	$-$ 3.17	$-$ 1.93
		$\sigma ^{2}_{b}$	11.4	$-$ 5.07	$-$ 4.98	9.01	$-$ 7.39	$-$ 5.58
		$\sigma _{bb}$	$-$ 55.6	$-$ 3.84	2.40	$-$ 36.4	$-$ 8.32	$-$ 3.28
	1000	$\mu$	$-$ 2.19	$-$ 0.91	$-$ 0.77	$-$ 3.95	$-$ 2.68	$-$ 0.75
		$\sigma ^{2}_{b}$	2.96	$-$ 0.95	$-$ 2.01	$-$ 1.73	$-$ 2.95	$-$ 2.53
		$\sigma _{bb}$	$-$ 33.3	$-$ 3.77	$-$ 0.55	$-$ 19.4	$-$ 6.28	$-$ 5.96
	2000	$\mu$	0.94	$-$ 0.14	$-$ 0.32	$-$ 0.25	$-$ 1.99	$-$ 0.09
		$\sigma ^{2}_{b}$	4.52	$-$ 1.55	$-$ 3.05	$-$ 3.67	$-$ 4.03	$-$ 3.44
		$\sigma _{bb}$	$-$ 3.67	6.63	4.20	0.82	3.69	$-$ 0.25
Bayes	–	$\mu$	$-$ 2.18	$-$ 2.59	$-$ 2.19	$-$ 2.69	$-$ 3.34	$-$ 1.99
		$\sigma ^{2}_{b}$	1.73	1.83	2.51	$-$ 1.44	1.44	1.13
		$\sigma _{bb}$	$-$ 55.7	$-$ 16.5	$-$ 9.31	$-$ 33.4	$-$ 11.3	$-$ 8.91

AGHQ = Adaptive Gauss–Hermit quadrature with 3, 4, or 5 nodes; QMC = Quasi Monte Carlo integration with 500, 1000, or 2000 points.

In the following, we drop the Laplace approximation from further consideration when we discuss the precision of the ML estimates (i.e., RB) and the confidence intervals (i.e., CR). Furthermore, to facilitate the interpretation of the results, we decided to average the results for the indices per parameter group (i.e., for the cognitive process parameter mean values *c*, *r*, *u*, and *a*, called $\mu$; the variance parameters in $\varvec{\Sigma }$, termed $\sigma _{b}^{2}$; and the covariance parameters in $\varvec{\Sigma }$, termed $\sigma _{bb}$). The resulting values for the relative bias are displayed in Table 2. When we consider relative biases below 10% to be acceptable and biases of 10–20% to be substantial (Forero et al., 2009; Morris et al., 2019), we found for both ML methods that relative biases decreased as the numbers of node vectors, sample sizes, and numbers of responses per participant increased. Importantly, relative biases were generally low and acceptable in all simulation conditions for the cognitive process parameters and the variance parameters. For the latter, however, biases were somewhat larger but still acceptable in case of AGHQ when the number of responses was 25 and the number of persons was 75. For the covariance parameters, the relative biases were larger and more substantial the smaller the number of points used and the smaller the number of responses. For the Bayes estimator, the relative bias was negligible for the cognitive process parameters and the variance parameters in all conditions. However, replicating the results of Klauer (2010), the covariance parameters were unacceptably and substantially biased when the number of responses was 25 or 75. When the number of responses was 125, the relative biases were still large but acceptable.

**Table 3:** Coverage rate of parameter estimates, depending on the approximation method, the number of individuals *T*, and the number of responses *N* per individual.

Method	No. Points	Parameter	*T* = 75			*T* = 125		
			*N* = 25	*N* = 75	*N* = 125	*N* = 25	*N* = 75	*N* = 125
AGHQ	$4^3$	$\mu$	84.5	92.3	92.9	87.8	93.9	94.3
		$\sigma ^{2}_{b}$	79.7	89.8	91.1	75.3	90.3	91.4
		$\sigma _{bb}$	85.1	91.6	94.5	83.3	91.6	94.3
	$4^4$	$\mu$	88.7	94.3	93.7	92.1	94.7	94.8
		$\sigma ^{2}_{b}$	88.1	90.9	92.4	88.5	93.0	92.8
		$\sigma _{bb}$	86.9	94.0	95.1	89.6	93.8	95.2
	$4^5$	$\mu$	94.6	94.6	93.9	93.9	95.0	94.8
		$\sigma ^{2}_{b}$	96.7	93.4	92.9	93.1	94.0	93.0
		$\sigma _{bb}$	93.4	95.5	95.4	92.7	94.1	95.2
QMC	500	$\mu$	77.9	89.0	92.4	80.1	89.3	94.0
		$\sigma ^{2}_{b}$	71.6	83.2	88.1	78.1	83.7	88.7
		$\sigma _{bb}$	68.0	85.5	92.7	74.9	86.6	93.2
	1000	$\mu$	81.8	91.9	93.4	78.9	93.4	94.6
		$\sigma ^{2}_{b}$	74.9	86.3	90.5	79.6	88.4	91.1
		$\sigma _{bb}$	66.4	89.8	94.2	76.6	90.3	94.3
	2000	$\mu$	86.7	92.2	93.5	84.5	93.6	94.5
		$\sigma ^{2}_{b}$	77.3	86.7	90.5	77.4	90.1	91.1
		$\sigma _{bb}$	83.2	90.5	94.4	82.1	90.3	94.2
Bayes	–	$\mu$	94.0	94.3	94.5	95.4	94.9	94.8
		$\sigma ^{2}_{b}$	95.1	94.3	94.7	95.9	94.7	95.3
		$\sigma _{bb}$	98.8	96.6	97.3	98.4	96.0	96.0

AGHQ = Adaptive Gauss–Hermit quadrature with 3, 4, or 5 nodes, QMC = Quasi Monte Carlo integration with 500, 1000, or 2000 points.

With regard to the CR (see Table 3), we found that for all three types of parameters, the CR moved closer to the nominal value as the sample size and the number of responses increased. For AGHQ, the CR was near its nominal value when the number of points was $4^4$ and the number of responses at least 75. When the number of responses is 25, the CR was near the nominal value when $4^5$ points were used. A similar pattern of results was observed for the QMC approximation, although the amount of undercoverage was greater than for the AGHQ approximation. Furthermore, when the number of responses was 25, undercoverage occurred irrespective of how many points were investigated in our simulation. In case of Bayes, we found that the coverage was nominal for the cognitive process parameters and the variance parameters. However, overcoverage occurred for the covariance parameters although this tendency disappeared the larger the number of responses.

To summarize, the simulation study reveals that the AGHQ method works better than the QMC method when the number of nodes is at least four. Relative biases were low even for small sample sizes and few responses per participant and confidence interval estimates were close to nominal. When the number of responses was 75, the QMC also provided acceptable results, at least when the number of points was 1000. Finally, when the number of responses was small (i.e., *R* = 25), AGHQ with five nodes yield estimates that are at least as good as the estimates of a Bayesian approach.

## Illustrative Example

To illustrate the proposed ML approach, we analyzed neuropsychological data from Schilken (1998), who employed the pair-clustering model to analyze and compare the memory performance of 22 epileptic patients with a right-temporal focus of epileptic seizures, 21 epileptic patients with a left-temporal focus, and 20 healthy controls matched with respect to age and intelligence. Each participant learned word lists consisting of 10 semantically strongly related word pairs (e.g., armchair-sofa) and 5 singletons that were not related to other words in the list (e.g., sunflower). All words were comparable in terms of difficulty and memorizability when considered in isolation. In addition, three words were added to the beginning and another three words to the end of the word list to absorb primacy and recency effects in free recall. Because these primacy and recency buffer words were excluded from further analysis, $N = 15$ responses per participant and study-test cycle remained for analysis-10 responses for the pairs and 5 for the singletons. The studying of the word list and the subsequent free recall test were repeated 6 times to examine learning effects on the *c*, *r*, and *u* parameters of the pair-clustering model, resulting in a total of six study-test trials per participant.

The same data set was also analyzed by Klauer (2006, 2010), thus providing us with the opportunity to compare our results with Klauer’s, which were based on the Bayesian Latent-Trait model (cf. Klauer, 2010, pp. 86/87). To maximize heterogeneity between participants, we followed the procedure outlined by Klauer (2006, 2010) and analyzed all $T = 22 + 21 + 20 = 63$ participants conjointly in our first model. Also following Klauer’s guidelines, we restricted our attention to the first two study-test cycles for each participant. This left us with $T = 63$ participants, $K = 4$ category systems (i.e., those for word pairs and singletons in Trials 1 and 2) with $J_1 = J_3 = 4$ and $J_2 = J_4 = 2$ categories (for word pairs and singletons, respectively), and $N_1 = N_3 = 10$ as well as $N_2 = N_4 = 5$ responses per participant within the four category systems. Finally, again in line with Klauer (2010)’s suggestions, we imposed the restriction that unclustered words in a pair must match the singletons in terms of trial-specific storage and retrieval probabilities (i.e., $u = a$). Hence, there were three parameters to estimate per trial, $c^{(1)}$, $r^{(1)}$, $u^{(1)}$ for Trial 1 and $c^{(2)}$, $r^{(2)}$, $u^{(2)}$ for Trial 2.

**Table 4:** Parameter estimates of the mean structure for the illustrative data example.

	Model 1				Model 2			
	Est	CI	TE	Bayes	Est	CI	$\Delta _{D.}$	TE
$\mu _{c^{(1)}}$	$-$ 0.58	[$-$ 0.87, $-$ 0.27]	0.28	0.31	$-$ 0.21	[$-$ 0.39, $-$ 0.03]		0.42
$\delta _{c^{(1)},D_1}$					$-$ 0.90	[$-$ 1.14, $-$ 0.64]	$-$ 1.11	0.13
$\delta _{c^{(1)},D_2}$					$-$ 0.47	[$-$ 0.71, $-$ 0.23]	$-$ 0.68	0.24
$\mu _{r^{(1)}}$	$-$ 0.33	[$-$ 0.68, 0.01]	0.37	0.35	$-$ 0.35	[$-$ 0.56, $-$ 0.14]		0.36
$\delta _{r^{(1)},D_1}$					0.16	[$-$ 0.01, 0.32]	$-$ 0.19	0.42
$\delta _{r^{(1)},D_2}$					0.10	[$-$ 0.06, 0.27]	$-$ 0.25	0.40
$\mu _{u^{(1)}}$	$-$ 0.93	[$-$ 1.06, $-$ 0.79]	0.18	0.18	$-$ 0.72	[$-$ 0.90, $-$ 0.53]		0.23
$\delta _{u^{(1)},D_1}$					$-$ 0.37	[$-$ 0.61, $-$ 0.12]	$-$ 1.09	0.14
$\delta _{u^{(1)},D_2}$					$-$ 0.30	[$-$ 0.56, $-$ 0.04]	$-$ 1.02	0.15
$\mu _{c^{(2)}}$	0.10	[$-$ 0.13, 0.34]	0.53	0.52	0.17	[ 0.02, 0.32]		0.57
$\delta _{c^{(2)},D_1}$					$-$ 0.12	[$-$ 0.40, 0.14]	$-$ 0.29	0.39
$\delta _{c^{(2)},D_2}$					$-$ 0.31	[$-$ 0.59, $-$ 0.04]	$-$ 0.48	0.32
$\mu _{r^{(2)}}$	$-$ 0.06	[$-$ 0.25, 0.12]	0.47	0.50	0.28	[$-$ 0.08, 0.64]		0.61
$\delta _{r^{(2)},D_1}$					$-$ 0.55	[$-$ 0.98, $-$ 0.13]	$-$ 0.83	0.20
$\delta _{r^{(2)},D_2}$					$-$ 0.43	[$-$ 0.91, 0.06]	$-$ 0.71	0.24
$\mu _{u^{(2)}}$	$-$ 0.36	[$-$ 0.51, $-$ 0.21]	0.36	0.34	$-$ 0.35	[$-$ 0.55, $-$ 0.15]		0.36
$\delta _{u^{(2)},D_1}$					$-$ 0.13	[$-$ 0.40, 0.12]	$-$ 0.48	0.32
$\delta _{u^{(2)},D_2}$					$-$ 0.07	[$-$ 0.32, 0.17]	$-$ 0.42	0.34

Parameters were obtained with AGHQ with 4 nodes. Est = Parameter estimates; CI = confidence interval; TE = probability-transformed estimates; Bayes = Bayesian Estimates as reported in Table 2 in Klauer (2010). D1 = First dummy variable, that is, 1 for right-temporal epileptic patients (all other participants are coded zero). D2 = Second dummy variable, that is, 1 for left-temporal epileptic patients (all other participants are coded zero). $\Delta _{D.}$ is the estimate of the parameter for the group coded in the respective dummy variable (i.e., $\delta _{.,D_{.}} + \mu _{.}$). For model 2, probability transformed estimates (column TE) indicate parameter means in the three groups (control group, right-temporal epileptics, left-temporal epileptics, respectively).

To test the goodness of fit, we estimated the model that we just specified and a more general model with four parameters $c^{(d)}$, $r^{(d)}$, $u^{(d)}$, and $a^{(d)}$ per trial. For both models, we used the AGHQ approximation method with 4 nodes to fit the two models. The LR test statistic comparing the original and the more general model was *LR* = 9.02, which is not significantly different from zero, $\chi ^{{2}}_{{crit}}$ = 30.1, *p* =.98, *df* = 19. Table 4 shows the parameters of the mean structure (i.e., $\mu$) in the restricted model. As can be seen, parameter values increased from Trial 1 to Trial 2, and the probability-transformed parameters were very similar to the probability-transformed parameters reported in Klauer (2010). Variance and correlation parameters were also quite similar across the two approaches (see Table 5). A notable exception was the variance of $r^{(1)}$, where the ML estimate was considerably larger than the Bayesian estimates. Also, the correlations involving this parameter were smaller for ML compared with Bayes.

**Table 5:** Parameter estimates of the covariance structure for the illustrative example with real data.

	Variances		Correlations					
	Est	Bayes	1.	2.	3.	4.	5.	6.
1. $c^{(1)}$	0.37	0.35	–	0.54	0.73	0.78	0.72	0.71
2. $r^{(1)}$	0.31	0.10	0.25	–	0.55	0.69	0.60	0.64
3. $u^{(1)}$	0.12	0.06	0.73	0.22	–	0.72	0.70	0.78
4. $c^{(2)}$	0.32	0.34	0.76	0.23	0.66	–	0.69	0.75
5. $r^{(2)}$	0.13	0.09	0.71	0.22	0.62	0.65	–	0.69
6. $u^{(2)}$	0.14	0.15	0.95	0.29	0.83	0.87	0.83	–

The first two columns present variance estimates based on the AGHQ method with 4 nodes (Est) and the corresponding Bayesian estimates (Bayes) as reported by Klauer (2010, Table 2), respectively. The correlation matrix displays the Bayesian estimates of Klauer (2010) above the diagonal and the corresponding AGHQ estimates using 4 nodes below the diagonal.

We decided to go one step further than Klauer (2010) and also analyze effects of the clinical group on the parameter estimates. For this purpose, we added two dummy variables as covariates to our model, the first one coded “1” for the right-temporal epileptic patients (patients in the two other groups were coded 0) and the second dummy variable coded “1” for the left-temporal epileptic patients (again, all other patients were coded “0”). This model provided us with the opportunity to estimate the average (negative) effect of each clinical group relative to the control group on each of the model parameters, that is, $c^{(1)}$, $r^{(1)}$, $u^{(1)}$ for Trial 1 and $c^{(2)}$, $r^{(2)}$, $u^{(2)}$ for Trial 2. The results are also shown in Table 4. The results indicate that estimates for the $c^{(.)}$ parameters were lower for epileptic patients compared with control patients whereas this pattern is less clear for the remaining parameters.

## Discussion

The aim of this article was to describe how the parameters in the latent-trait MPT model can be estimated with a marginal ML approach. Specifically, we introduced three methods to approximate the integrals that are involved when the goal is to maximize the marginal log-likelihood function, and we investigated the statistical properties of these methods in a simulation study. Finally, we presented an empirical example that illustrated the suggested approaches.

The results of the simulation study showed that AGHQ and QMC performed well with regard to the relative bias and the coverage rate. However, we also found that AGHQ performed somewhat better than QMC in most simulation conditions. We would therefore recommend that researchers use AGHQ as the default method for parameter estimation but switch to QMC when the number of random effects becomes too large to approximate the integral with AGHQ in a reasonable amount of time. Strictly speaking, however, our results refer to the range of simulation conditions examined here only. Hence, further simulation research is needed to examine the performance of our ML approaches for other MPT models or, for example, models with low variance components. In these simulation studies, one could then also examine some additional approximation methods we ignored in the current study, such as a Monte Carlo EM algorithm (Booth and Hobert, 1999), variational approximation (Ormerod and Wand, 2010), or Laplace importance sampling (Kuk, 1999). The latter method is a modification to the Laplace approximation which we dropped from further consideration because of unsatisfactory performance in our simulation study. Laplace importance sampling is an interesting alternative that is definitely worth to investigate.

So far, the parameters of the latent-trait MPT model can be estimated with a Bayesian approach only (Heck et al., 2018a; Klauer, 2010). Our simulation study suggests that the maximum likelihood approach introduced here-specifically, the AGHQ approximation method-provides estimates that are at least as good as Bayesian estimates (provided the number of nodes is sufficiently high). For covariance parameters, in particular, both relative estimation bias and coverage rates of confidence intervals appear to be clearly superior for AGHQ-based ML estimates compared to their Bayesian counterparts. These results are important for empirical applications, especially those focusing on parameter correlations. In addition, the maximum likelihood approach has some pragmatic advantages compared to the Bayesian approach, for example, because prior distributions are not required, the convergence of the estimation algorithm is easier to determine, and the asymptotic optimality properties of ML-estimated parameters have been well-known in the statistical literature for decades. This does not mean that we are rejecting a Bayesian approach; rather, we believe that the two approaches complement each other and that there likely are situations in which one approach is preferable to the other (Wasserman, 2004).

We believe that further simulation research is needed to determine the best-performing method for a variety of situations. For example, on the one hand, estimation of covariances between MPT parameters may turn out to be a specific strength of ML methods. On the other hand, the Bayesian approach may outperform ML methods when the number of participants and/or the number of responses is small. In fact, the results of our simulation study suggest these tentative interpretations. However, our results require replication and definitely need to be extended to other MPT models before they can provide a basis for general recommendations. Furthermore, another interesting question for future research is whether there are circumstances under which the two methods produce discrepant results concerning model comparisons. Finally, we also think that it is interesting to investigate whether a combination of the two estimators (e.g., using the ML estimates as starting values for Bayesian MCMC estimation) has better asymptotic properties compared to each single approach alone.

Additionally, there are a number of further research questions that we think would be worthwhile to study. First, it would be interesting to extend the ML estimator proposed here to handle both random participant and random item effects (Matzke et al., 2015). Implementing such a crossed random effects MPT model would be a challenging task for future research. Another challenging issue concerns recent extensions of MPT models to include continuous variables such as response times (Heck et al., 2018b; Klauer and Kellen, 2018). These generalized MPT models could also be embedded in a hierarchical random effects framework and analyzed using the marginal ML methods proposed here.

A major problem involves convergence problems of marginal ML methods, for example, when the number of responses per participant is very small or when the true variance components are small. It would be interesting to investigate whether a penalized maximum likelihood estimator (Chung et al., 2013) can solve the convergence issues of the ML estimator. Finally, both the Bayesian approach and the ML approach proposed here assume that the random effects are multivariate normally distributed. From a statistical point of view, however, this assumption does not need to be true, and it would be interesting to examine how robust the two approaches are with respect to such a misspecification when the true underlying distribution is actually, for example, a finite mixture distribution (a reasonable assumption for the illustrative example). We note that one can specify arbitrary distributions for the random effects in ML with QMC sampling and that the implementation of these “robust” ML estimators would also be interesting for future research.

In summary, the present article shows how marginal maximum likelihood estimation can be used to obtain the parameters of a random effects MPT model with or without covariates. Using the pair-clustering model as a running example, we found for both simulated and real data that the ML approach is a reasonable alternative to Bayesian hierarchical MPT analyses that are based on the Latent-Trait Model (Heck et al., 2018a; Klauer, 2010). Future research should extend these results to other, more complex MPT models and perhaps also explore alternative numerical methods of marginal ML estimation.

## Footnotes

1 We used the numerically approximated Hessian matrix to compute the standard errors of the parameter estimates in a replication. Additional analyses with 100 replications from the simulation condition with 75 participants and 25 responses showed that differences between standard errors based on the numerically approximated Hessian and the analytically computed Hessian were very small. Therefore, we conclude that all unsatisfactory results regarding the coverage rate are not due to the numerical approximation of the Hessian matrix.
